# Integrin-Linked Kinase Activation Prevents Ventricular Arrhythmias Induced by Ischemia/Reperfusion Via Inhibition of Connexin 43 Remodeling

**DOI:** 10.1007/s12265-020-09979-2

**Published:** 2020-03-06

**Authors:** Ping Zhou, Xiaoli Yang, Dezhong Yang, Xin Jiang, Wei Eric Wang, Rongchuan Yue, Yuqiang Fang

**Affiliations:** 1 Department of Cardiology, The First People’s Hospital of Chongqing Liang Jiang New Area, Chongqing, 401121 China; 2 Department of Cardiology, Chongqing Institute of Cardiology, Daping Hospital, Army Medical University, 10 Changjiang Branch Road,Yuzhong District, Chongqing, 400042 China

**Keywords:** Integrin-linked kinase, Ischemia reperfusion, Ventricular arrhythmia, Connexin 43

## Abstract

Ischemia reperfusion (I/R)-induced arrhythmia is a serious complication in patients with cardiac infarction. Remodeling of connexin (Cx) 43, manifested as phosphorylation, contributes significantly to arrhythmogenesis. Integrin-linked kinase (ILK) attenuated ventricular remodeling and improved cardiac function in rats after myocardial infarction. We hypothesized that ILK, through Cx43 phosphorylation, would be protective against I/R-induced ventricular arrhythmias. Our study showed that I/R-induced ventricular arrhythmias were attenuated by an ILK agonist LPTP and worsened by the ILK inhibitor Cpd22. I/R disrupted Cx43 distribution, but it was partially normalized in the presence of LPTP. Compared with I/R, the phosphorylation of Akt was increased significantly after pretreatment with LPTP. The increase in phosphorylated Akt was physiologically significant because, in the presence of the Akt inhibitor MK2206, the protective effects of LPTP were blocked. This indicated that ILK activation prevented I/R-induced-ventricular arrhythmia, an effect potentially related to inhibition of Cx43 remodeling via Akt activation.

## Introduction

Ischemic heart disease is among the most prevalent worldwide health problems [1]. It is caused primarily by coronary occlusion, which leads to tissue hypoxia, cellular necrosis, and apoptosis. Early reperfusion after coronary occlusion is proven as the most efficient way to improve cardiac function and decrease infarct size. The sooner the reperfusion, the better the outcome [2]. Unfortunately, reperfusion is invariably accompanied by additional injury, in the form of myocyte death, endothelial and microvascular dysfunction, and fatal reperfusion arrhythmias, such as ventricular tachycardia (VT) and ventricular fibrillation (VF) [3–5]. Although reperfusion arrhythmia is an important marker of successful reperfusion of an occluded coronary artery, it is also a major clinical manifestation leading to cardiac death. To overcome the effects of severe ventricular arrhythmias in ischemia-reperfusion (I/R) injured hearts is a major challenge. Therefore, it is important to clarify the underlying mechanism(s) of I/R-induced cardiac arrhythmia.

Integrin-linked kinase (ILK) is a 59-kDa serine/threonine protein kinase that is highly expressed in a variety of mammalian cells and tissues [6]. ILK is linked to cell–matrix interactions through its binding to the cytoplasmic domain of integrins. It regulates several downstream intracellular signaling systems, including the Akt, glycogen synthase kinase 3β, extracellular signal-regulated kinases, myosin light chain, and Rac1 pathways [7–9]. In this manner, it modulates cytoskeletal remodeling and biological processes, such as proliferation, growth, differentiation, invasion, and survival [10]. ILK is highly expressed, serving dual functions as a mechanoreceptor and a nodal regulator of adaptive, prohypertrophic signaling in the postnatal heart [11–13]. Knockout of the ILK gene in the murine heart induced dilated cardiomyopathy and spontaneous heart failure [14]. Transfection with the ILK gene attenuated left ventricular remodeling and improved cardiac function in rats after myocardial infarction [15], while also improving cardiac function and decreasing mortality in a model of dilated cardiomyopathy [16]. ILK deletion in cardiomyocytes produced a lethal arrhythmogenic cardiomyopathy [17], suggesting that ILK can participate in development of arrhythmias. However, whether ILK can prevent I/R-induced ventricular arrhythmias is unknown. Cx43 remodeling was demonstrated in I/R-induced arrhythmias, while Traister et al. found that, in human fetal myocardial cells (19–22 weeks gestation), downregulation of endogenous ILK by siRNA led, in parallel, to decreased Cx43 expression [18]. These results implied that ILK was involved in regulating Cx43. Thus, we hypothesized that ILK would inhibit I/R-induced ventricular arrhythmias. Our study used a Langendorff-based ex vivo system.

## Materials and Methods

### Animal Preparation

The study conformed to the Guide for the Care and Use of Laboratory Animals published by the US National Institutes of Health (Bethesda, MD; NIH Publication No. 85–23, revised 1996). All animal (*n* = 61) experiments were approved by the Animal Use and Care Committee of Daping Hospital. Male Sprague-Dawley rats, weighing between 200 and 230 g, were approved for use by the Daping Hospital Laboratory Animal Center.

### Langendorff-Based Ex Vivo Model of Cardiac Ischemia/Reperfusion

The rats were anesthetized with pentobarbital sodium (intraperitoneal (ip), 50 mg/kg) after ip administration of heparin sodium (1000 IU/kg). The hearts were rapidly excised and mounted onto the Langendorff perfusion system with ascending aortic cannulation, then perfused with an oxygenated (95% O_2_–5%CO_2_) equilibrated KHS(Krebs-Henseleit solution) containing (in mM) 118.5 NaCl, 4.7 KCl, 1.2 MgSO_4_, 1.8 CaCl_2_, 24.8 NaHCO_3_, 1.2 KH_2_PO_4_, and 10 glucose, pH 7.4, at 37 °C, as described previously [19].

The left anterior descending (LAD) coronary artery was ligated using a (6-0) polypropylene suture. The artery was occluded for 30 min to induce regional ischemia, followed by 30 min reperfusion. In sham procedures, the suture was placed around the artery but not tightened. Hearts demonstrating contractile abnormalities, more than 5 ventricular premature beats (VBPs), ventricular tachycardia (VT), or ventricular fibrillation (VF) appearing during the stabilization period or an absence of signs of successful coronary artery occlusion were removed from the study. Surface ECGs were obtained from the right atrium and left ventricle, recorded in digital format and analyzed with a PowerLab system (A.D. Instrument, New South Wales, Australia).

### Experimental Protocol

All hearts were subjected to a 20-min stabilization period, 30-min regional ischemia, and 30-min reperfusion. The hearts were randomly divided into individual groups, a sham group, I/R group, and I/R with reagent treatment group. In the sham group, the suture was not tightened and hearts were perfused constantly with KHS for 80 min without any treatment. In the I/R group, the hearts were stabilized for 20 min, then the LAD was occluded for 30 min to induce regional ischemia, followed by 30-min reperfusion with KHS. The reagents included ILK agonist L-α-phosphatidyl-D-myo-inositol 3,4,5-triphosphate,dioctanoyl (LPTP) (10^−7^ M) (Sigma, St. Louis, MO), ILK inhibitor Cpd22 (6 × 10^−7^ M) (Millipore, St Charles, MO), and the Akt inhibitor MK2206 (7 × 10^−8^ M) (Selleckchem, Houston, TX). Reagents were dissolved in KHS and infused before inducing ischemia. When an inhibitor and agonist were infused into the same isolated heart, the inhibitor was introduced 10 min before the agonist.

### Analysis of Arrhythmias

The incidence and severity of ventricular arrhythmias during reperfusion were characterized in accordance with the guidelines of the Lambeth Convention [20]. Isolated VBPs were defined as discrete and identifiable premature QRS complexes. VT was defined as a run of four or more consecutive ventricular premature beats. VF was defined as a signal for which individual QRS deflections could no longer be distinguished from one another (implying morphological instability) and for which a rate could no longer be measured. The VF which can be terminated spontaneously in 2 min was defined as SVF, then the VF which could not be terminated spontaneously in 2 min was called NVF. The ventricular arrhythmias were scored using the Arrhythmia Score described by Curtis and Walker [21] (Table 1).

**Table 1:** Arrhythmia scoring system

Score	The type of ventricular arrhythmias
0	0–20 VBPs
1	21–100 VBPs
2	> 100 VPBs or 1–3 episodes of VT or both
3	> 3 episodes of VT
4	SVF
5	1 or 2 episodes of NVF
6	3–5 episodes of NVF
7	> 5 episodes of NVF

Exclusion criteria: Experiments were terminated or excluded from the final data analysis, if any of the following conditions occurred: more than 5 VBPs, VT or VF appeared during stabilization, absence of signs of successful coronary artery occlusion, or severe atrioventricular block during the first 5 min ischemia

### Immunoblotting

Proteins were separated by sodium dodecylsulfate–polyacrylamide gel electrophoresis (SDS–PAGE) and transferred to nitrocellulose membranes. Nonspecific proteins were blocked with 5% nonfat dried milk in Tris-buffered saline Tween (TBST) buffer for 1 h at room temperature with agitation and then membranes were incubated with various primary antibodies. These were monoclonal mouse anti-Cx43 (1:1000; Invitrogen, Carlsbad, CA), monoclonal rabbit anti-Akt (1:1000; Cell Signaling Technology, Beverly, MA), phosphorylated Akt^473^ (1:1000; Cell Signaling Technology), or rabbit anti-GAPDH (1:500; Santa Cruz Biotechnology, Santa Cruz, CA) overnight at 4–8 °C. After washing 3 times for 10 min per times in TBST, each membrane was incubated with the homologous secondary antibody for 1 h at room temperature. The images were analyzed using Quantity One software (Bio-Rad, Hercules, CA) to obtain the integrated intensities. Each experiment was repeated five times.

### Immunofluorescence Staining

Rat heart samples were fixed in 4% paraformaldehyde and embedded in paraffin. Heart tissues were cut into 4–5 μm sections for immunofluorescence detection of Cx43. The polyclonal rabbit anti-Cx43 antibody (Millipore) was used at a dilution of 1:100 as the primary antibody and goat anti-rabbit IgG (Jackson ImmunoResearch, West Grove, PA) at a dilution of 1:200 as the secondary antibody. The cardiomyocytes were counterstained with DAPI (Beyotime, Jiangsu, China). Immunofluorescence was detected by immunofluorescence microscopy (Nikon, Tokyo, Japan). Cx-43 density measurements were analyzed by Image J software. Cx-43 remodeling was defined by the percentage of Cx-43 at the intercalated disks.

### Statistical Analysis

Except for the incidences of VT and VF, which were expressed as percentages, all other data were expressed as means ± SEM. To compare arrhythmia scores, one-way ANOVA was used for comparisons among groups and the LSD test was applied to test for differences between individual groups. Incidences of ventricular arrhythmias (VT and VF) were compared by Fisher’s exact test. Durations of VT and VF were analyzed with the Kruskal-Wallis test. *P* values of < 0.05 were considered statistically significant.

## Results

### Effects of ILK on I/R-Induced Ventricular Arrhythmias

**Fig. 1:**
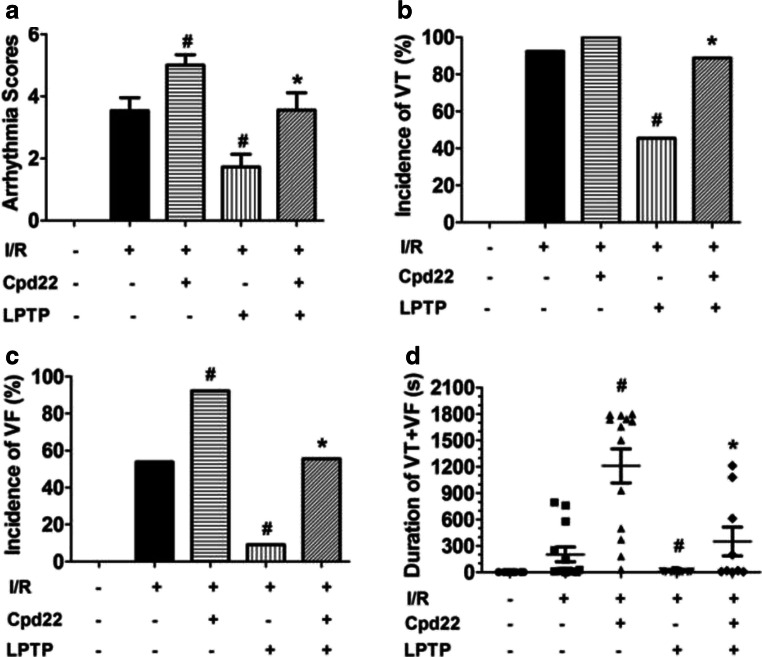
Effect of ILK on I/R-induced ventricular arrhythmias. The ILK agonist, LPTP (10^−7^ M), and the antagonist, Cpd22 (6 × 10^−7^ M), were administrated 10 min prior to ischemia. Arrhythmia score (**a**), incidences of VT (**b**) or VF (**c**), and duration of VT + VF (**d**) were recorded (*n* = 5–13, ^#^*P* < 0.05 vs I/R; *P < 0.05 vs I/R + LPTP)

**Fig. 2:**
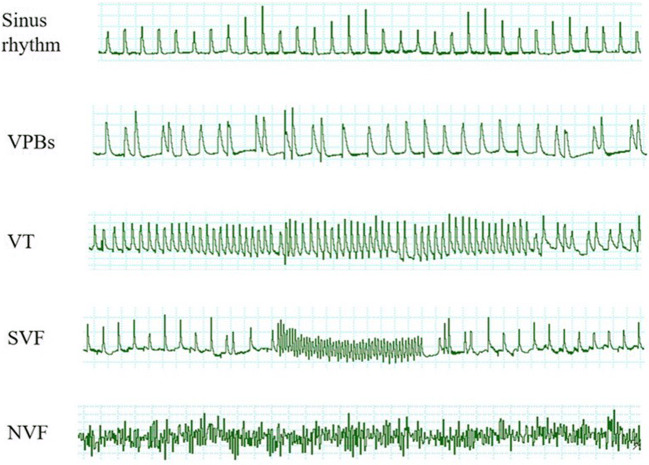
The representative typical arrhythmia after I/R. SVF: the VF which can be terminated spontaneously in 2 min; NVF: the VF which could not be terminated spontaneously in 2 min

Consistent with previous reports [3], during the 20-min stabilization period, none of the hearts developed VT or VF. In contrast, after reperfusion, ventricular arrhythmias, including VPBs, VT, or VF, occurred in most hearts. As shown in Fig. 1 a, compared with the I/R group, ILK agonist LPDT could significantly reduce the arrhythmia score (1.7 ± 1.3 vs 3.5 ± 1.5, *P* < 0.05). Contrastly, ILK inhibitor Cpd22 could increase the arrhythmia score after reperfusion (5.0 ± 1.2 vs 3.5 ± 1.5, *P* < 0.05); moreover, Cpd22 could abolish the effect of LPDT on arrhythmia score (3.6 ± 1.7 vs 1.7 ± 1.3, *P* < 0.05) (Fig. 1a). The VT (5/11, 45.5%) and VF (1/11, 9.1%) in the I/R + LPDT group were significantly decreased compared with those in the I/R group (12/13, 92.3%; 7/13, 53.8%; respectively, both *P* < 0.05). There was no significant difference in VT between the I/R group and the I/R + Cpd22 group (12/13, 92.3% vs 13/13, 100.0%; *P* > 0.05). However, in the I/R + Cpd22 group, the VF (12/13, 92.3%) was significantly increased compared with that in the I/R group (7/13, 53.8%) (*P* < 0.05). Administration of Cpd22 could block the protective effect of LPDT on VT (8/9, 88.9% vs 5/11, 45.5%, *P* < 0.05) and VF (5/9, 55.6% vs 1/11, 9.1%, *P* < 0.05) (Fig. 1 b and c). The duration of VT + VF in the I/R + LPDT group was significantly shorter than that in the I/R group (*P* < 0.05). In contrast, Cpd22 extended the duration of VT + VF compared with the I/R group (*P* < 0.05). In accordance with arrhythmia score and incidences of malignant arrhythmias, administration of Cpd22 could antagonize the protective effect of LPDT on duration of VT + VF (*P* < 0.05) (Fig. 1d). We showed the representative typical arrhythmia after I/R in Fig. 2.

### Role of Cx43 in the Protective Effects of ILK

**Fig. 3:**
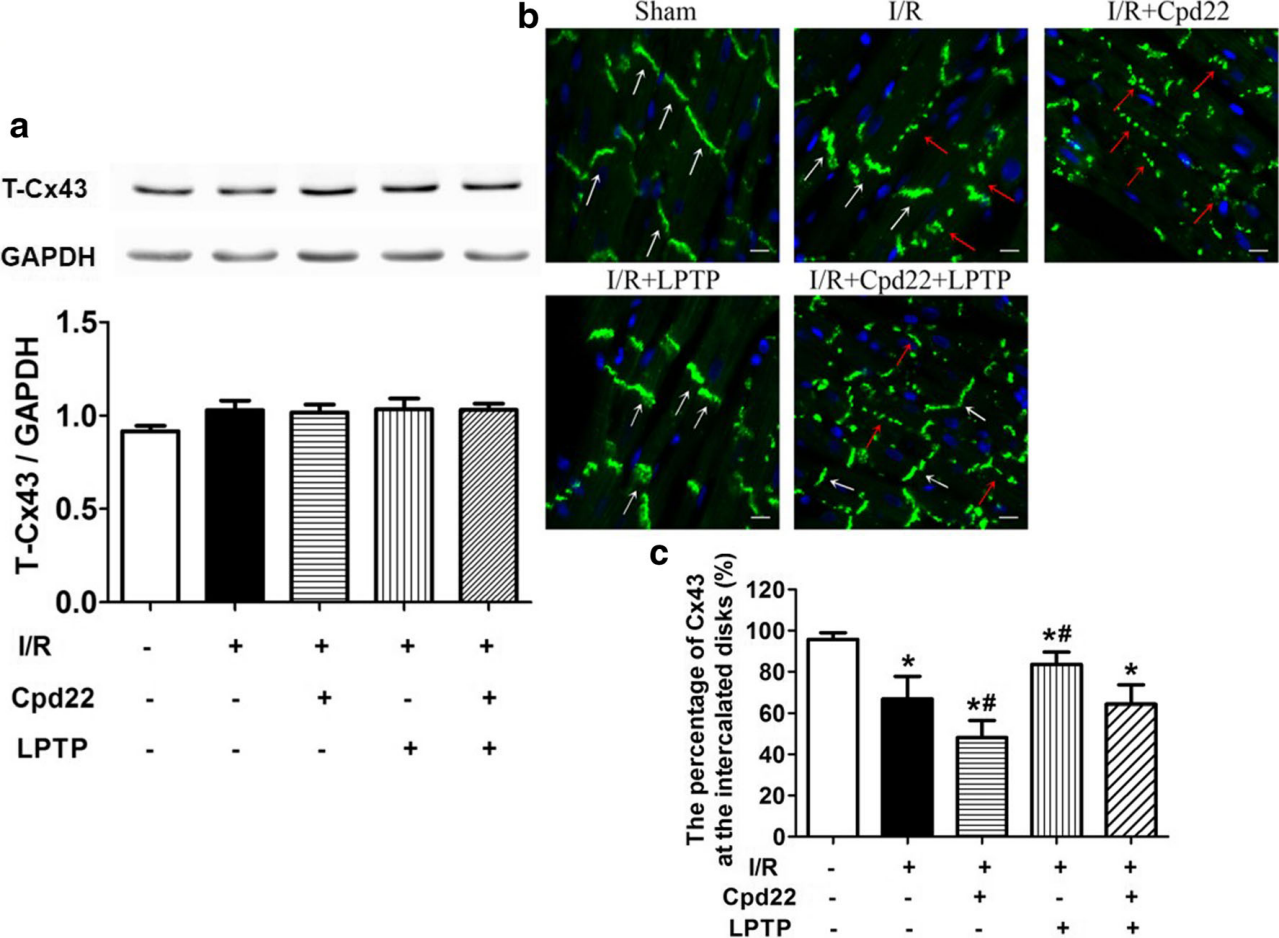
Effect of ILK on I/R-induced Cx43 remolding. LPTP and Cpd22 were administrated 10 min prior to ischemia. Total Cx43 expression was determined by immunoblotting (**a**) (*n* = 5, P=NS). The distribution of Cx43 was recorded by immunofluorescence microscopy (**b**) (*n* = 5). In the sham group, white arrows show representative Cx43 (green fluorescence) at the intercalated disks running across the longitudinal axis of the constituent myocytes. In the I/R group, some typical intercalated disk distribution of Cx43 were present (white arrows), while Cx43 in cytoplasm was increased and lateralization of Cx43 was also found (red arrows). In the I/R + LPTP group, Cx43 was mainly distributed at the intercalated disks, with less in the cytoplasm and lateralization. In the I/R + Cpd22 group, lateralization of Cx43 and Cx43 in the cytoplasm was increased (red arrows), intercalated disk distribution of Cx43 was scarcely found. In the I/R + Cpd22 + LPTP group, some Cx43 was distributed at the intercalated disks (white arrows), lateralization of Cx43 and Cx43 in cytoplasm was less than in the I/R + Cpd22 group (red arrows) (magnification ×40). To clarify the above results, we defined the percentage of Cx-43 at the intercalated disks as Cx-43 remodeling by measuring the Cx-43 density using Image J software (**c**) (^#^*P* < 0.05 vs I/R; *P < 0.05 vs sham; *n* = 5)

It was reported that Cx43 remodeling was essential for arrhythmogenesis [22, 23]. To determine whether the anti-arrhythmic effects of ILK were relevant to Cx43 remodeling, we examined Cx43 expression and distribution; also we quantified the expression of Cx43 at the intercalated disks. There were no significant differences among all groups in total Cx43 levels (Fig. 3a). Cx43 is normally highly organized into clusters at the intercalated disks running across the longitudinal axis of the constituent myocytes. Only low levels were located in the cytoplasm and the lateral interfaces of myocytes. I/R injury disrupted the normal distribution of Cx43. Thus, intercalated disk distribution was decreased, with more Cx43 distributed along the lateral interfaces between myocytes (called lateralization) and some observed in the cytoplasm of myocardiocytes. In the presence of LPTP, Cx43 distribution was partially normalized. In contrast, pretreatment with Cpd22 augmented the abnormal redistribution of Cx43. The protective effect of LPTP on Cx43 distribution was prevented in the presence of Cpd22, further supporting the specificity of the ILK agonist, LPTP, and the antagonist, Cpd22 (Fig. 3b, c).

### Effects of Akt on ILK-Mediated Anti-Arrhythmia

**Fig. 4:**
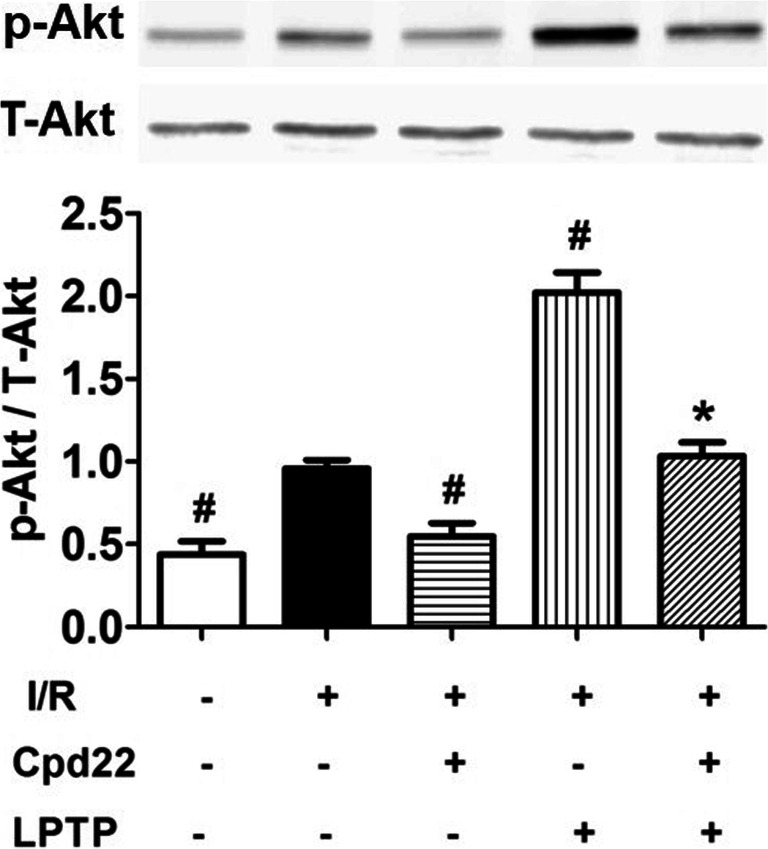
Effect of ILK on phospho-Akt^473^ expression in heart with I/R injury. LPTP and Cpd22 were administrated 10 min prior to ischemia; phospho-Akt^473^ expression was determined by immunoblotting (^#^*P* < 0.05 vs I/R; *P < 0.05 vs I/R + LPTP; *n* = 5)

Akt was shown to phosphorylate Cx43 on S373 [24]. Increased gap junction stability was associated with Akt-mediated Cx43 phosphorylation on S373 [25]. We examined phosphorylated Akt, its active form, by immunoblotting. Although total Akt levels were not changed, I/R increased Akt phosphorylation only slightly, but it was significantly increased by pretreatment with LPTP. The increase in phosphorylated Akt was physiologically significant, because its protective effects were blocked by the ILK inhibitor, Cpd22 (Fig. 4).

**Fig. 5:**
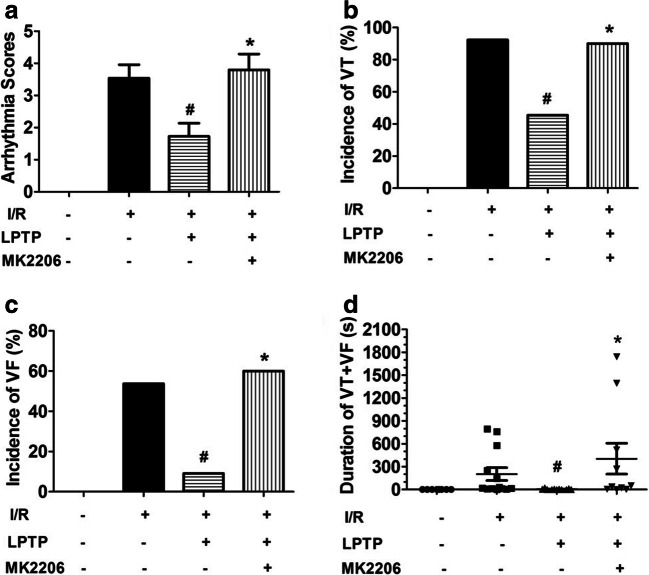
Effect of Akt on I/R-induced ventricular arrhythmias. The Akt inhibitor MK2206 (7 × 10^−8^ M) was administrated 10 min prior to pretreatment with the ILK agonist LPTP (10^−7^ M, 10 min) prior to ischemia. Arrhythmia score (**a**), incidences of VT (**b**) or VF (**c**), and duration of VT + VF (**d**) were recorded (*n* = 5–13, ^#^*P* < 0.05 vs I/R; *P < 0.05 vs I/R + LPTP)

**Fig. 6:**
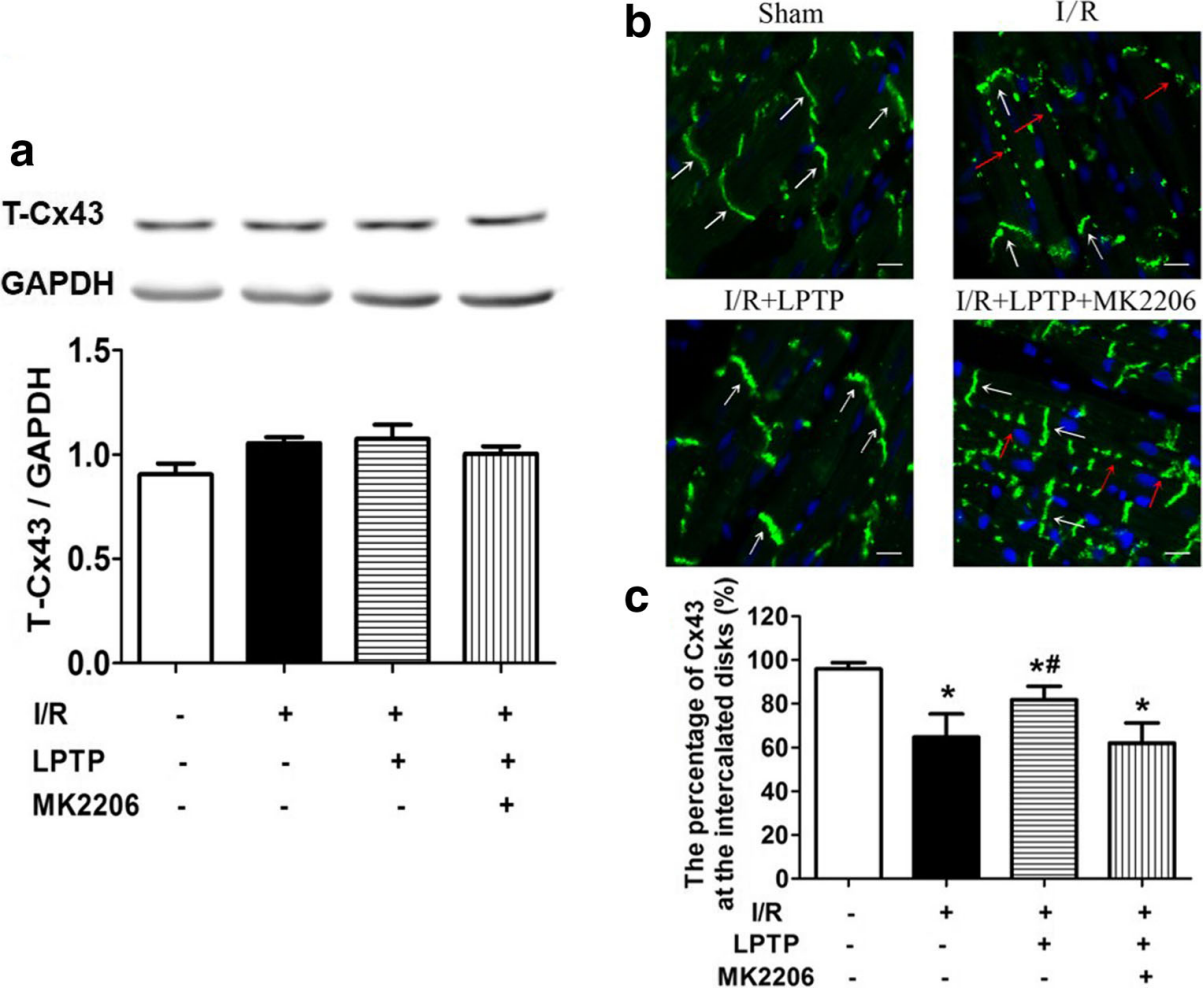
Effect of Akt on I/R-induced Cx43 remolding. The Akt inhibitor MK2206 (7 × 10^−8^ M) was administrated 10 min prior to pretreatment with the ILK agonist LPTP (10^−7^ M, 10 min) before ischemia. Total Cx43 expression was determined by immunoblotting (**a**) (*n* = 5, P=NS). The distribution of Cx43 was recorded by immunofluorescence microscopy with or without MK2206 treatment (**b**) (*n* = 5). In the I/R + MK2206 + LPTP group, Cx43 distributed at intercalated disks were found (white arrows), lateralization of Cx43 and Cx43 in cytoplasm was much more than in the I/R + LPTP group (red arrows) (magnification ×40). Cx-43 remodeling was defined by the percentage of Cx-43 at the intercalated disks; Cx-43 density measurements were analyzed by Image J software (**c**) (^#^*P* < 0.05 vs I/R; *P < 0.05 vs sham; *n* = 5)

**Fig. 7:**
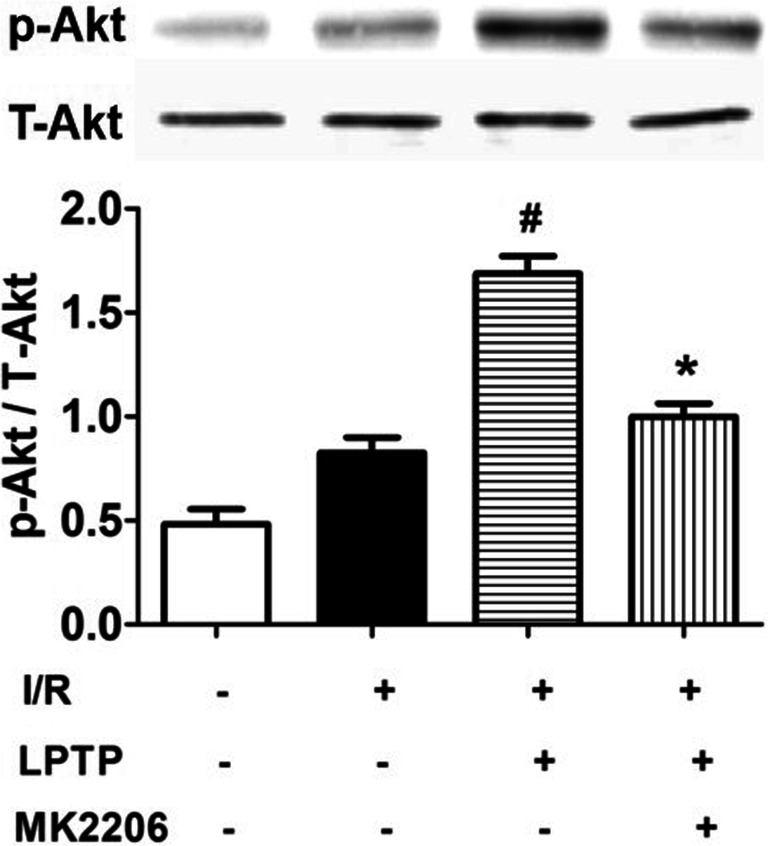
Effect of Akt on phospho-Akt^473^ expression in hearts with I/R injury. The Akt inhibitor MK2206 (7 × 10^−8^ M) was administrated 10 min prior to pretreatment with the ILK agonist LPTP (10^−7^ M, 10 min) before ischemia (^#^*P* < 0.05 vs I/R; *P < 0.05 vs I/R + LPTP; *n* = 5)

To further validate the role of Akt in ILK-mediated anti-arrhythmia, we perfused hearts with the Akt inhibitor MK2206 (7 × 10^−8^ M, 10 min) before administering the ILK agonist LPTP (10^−7^ M, 10 min), and analyzed the occurrence of arrhythmias. As shown in Fig. 5, the protective effects of LPTP were antagonized in the presence of the Akt inhibitor MK2206. Consistent with the findings illustrated in Fig. 3, there were no significant differences among groups in levels of total Cx43 (Fig. 6a). However, I/R injury redistributed the Cx43 in the heart and Cx43 distribution was partially normalized by LPTP, while MK2206 inhibited these effects of LPTP (Fig. 6b). Akt phosphorylation was further confirmed, with LPTP increasing levels of phosphorylated Akt in the I/R-injured heart, and this effect was also blocked by MK2206 (Fig. 7).

## Discussion

I/R arrhythmias are caused by reperfusion of the heart tissue, after coronary blood flow interruption, through natural re-opening of blood vessels, drug effects, or mechanical recanalization. Such arrhythmias are associated with changes in electrical activity induced by biochemical and physiological alterations. I/R arrhythmias can be used as indicators of coronary recanalization [26]. Reperfusion arrhythmias, which primarily occur in early reperfusion at a high incidence, up to 80%, after thrombolytic therapy for myocardial infarction, can lead to serious consequences, such as cardiac arrest and cardiogenic shock. Although the mechanism(s) are not clear, Cx43 remodeling, including changes in its expression, distribution, structure, and function, is believed to play an important role in I/R arrhythmias [27].

In the heart, gap junctions are important for normal function, such as electrical impulses and myocardial contractility, of cardiac muscle. These gap junctions promote electrical cell–cell coupling, that is, impulse propagation between cells, because their electrical resistance is much lower than that of the cell membrane [27]. It is known that regulation of gap junctions mainly depends on connexin, which forms the gap junction channel [27]. Remodeling of connexin is shown to be an essential step for arrhythmogenesis after I/R [22, 28, 29]. In normal rat ventricular myocytes, Cx43 is highly organized into clusters at the intercalated disks lying transverse to the longitudinal axis of the constituent myocytes. I/R could induce Cx43 gap junction remodeling, including downregulation of the protein, decreased gap junction plaque size, increased heterogeneity and lateralization of gap junction distribution, and dephosphorylation. Our study demonstrated lateralization of Cx43 in ventricular myocytes after I/R injury. Pretreatment with the ILK inhibitor Cpd22 increased lateralization of Cx43. In contrast, the ILK agonist, LPTP, inhibited I/R-induced Cx43 lateralization, thereby suppressing ventricular tachyarrhythmia.

ILK is associated with cardiac contractility [30], ventricular hypertrophy [31], and repair [11]. Targeted ILK ablation in the murine heart induces dilated cardiomyopathy and spontaneous heart failure [14]. Xu et al. found that overexpression of ILK, such as in ILK-overexpressing Sca-1^+^ cardiac progenitor cells, attenuated left ventricular remodeling and improved cardiac function in rats after myocardial infarction [15, 32]. ILK overexpression also improves cardiac function and decreased mortality in a model of dilated cardiomyopathy [16]. Using conditionally ILK-deficient mice and human atherosclerotic arteries, Herranz et al. reported that ILK regulated vasomotor function by preventing endothelial nitric oxide synthase uncoupling, which is involved in atherosclerosis [33]. Other reports show that ILK downregulation by siRNA in cardioblasts is accompanied by a parallel decrease in expression of the cardiomyocyte gap junction protein Cx43 [18]. In our study, ILK activation prevented I/R-induced ventricular arrhythmias.

Consistent with previous reports [34], the expression of p-Akt was increased during ischemia-reperfusion, and our study found that LPTP, an agonist of ILK, can further increase the expression of p-Akt, while reducing the occurrence of reperfusion arrhythmias, and this protection was eliminated by the p-Akt antagonist MK2206. The increasing of p-Akt during ischemia-reperfusion is caused by the body’s self-protection, but the increasing is not sufficient to counteract the damage caused by ischemia-reperfusion, but when LPTP was present, the expression of p-Akt was significantly increased which can reduce reperfusion arrhythmias.

Akt is shown to phosphorylate Cx43 on S373 [24]. Increased gap junction stability is associated with Akt-mediated Cx43 phosphorylation on S373 [25]. Inhibition of Akt, with either specific chemicals or dominant negative constructs, leads to functional loss of gap junctions. Phosphorylation of Cx43 at S373 by Akt is reported to potentially control gap junction size through inhibition of Cx43 and ZO-1 interactions [35]. In our study, ILK prevented I/R-induced ventricular arrhythmias. While, in the presence of Akt inhibitor, MK2206, the protective effects of the ILK agonist, LPTP, on normalization of Cx43 distribution and ventricular arrhythmia were prevented. The mechanisms leading to ILK phosphorylation of Akt are complex, involving both direct and indirect signaling pathways. In a study using an in-gel kinase assay and matrix-assisted laser desorption-ionization time-of-flight mass spectrometry, purified ILK directly phosphorylates PKB/Akt. Co-immunoprecipitation analysis of cell extracts demonstrated that ILK formed a complex with PKB/Akt, as well as with PDK-1, and that ILK disrupted PDK-1/PKB associations [36]. Lynch et al. reported that ILK promoted PKB/Akt-induced serine 473 phosphorylation indirectly, via an adapter function [37]. Our findings further confirmed regulation of ILK by Akt phosphorylation in the I/R-injured heart.

## Conclusions

Our study showed that ILK activation prevented I/R-induced-ventricular arrhythmias, an effect potentially related to inhibition of Cx43 remodeling, through Akt activation. These findings indicated that ILK is a potential therapeutic target for preventing reperfusion-associated ventricular arrhythmias.

## Abbreviations

Cx43: Connexin (Cx) 43
ILK: Integrin-linked kinase
I/R: Ischemia reperfusion
LAD: left anterior descending
LPTP: L-α-phosphatidyl-D-myo-inositol 3,4,5-triphosphate,dioctanoyl
KHS: Krebs-Henseleit solution
VBPs: Ventricular premature beats
VT: Ventricular tachycardia
VF: Ventricular fibrillation
